# Procedural-Memory, Working-Memory, and Declarative-Memory Skills Are Each Associated With Dimensional Integration in Sound-Category Learning

**DOI:** 10.3389/fpsyg.2018.01828

**Published:** 2018-10-02

**Authors:** Carolyn Quam, Alisa Wang, W. Todd Maddox, Kimberly Golisch, Andrew Lotto

**Affiliations:** ^1^Department of Speech and Hearing Sciences, Portland State University, Portland, OR, United States; ^2^Department of Speech, Language, and Hearing Sciences, University of Arizona, Tucson, AZ, United States; ^3^Department of Psychology, University of Arizona, Tucson, AZ, United States; ^4^Cognitive Design and Statistical Consulting, LLC., Austin, TX, United States; ^5^College of Medicine–Tucson, University of Arizona, Tucson, AZ, United States; ^6^Department of Speech, Language, and Hearing Sciences, University of Florida, Gainesville, FL, United States

**Keywords:** speech-sound learning, language acquisition, individual differences, memory, cognition

## Abstract

This paper investigates relationships between procedural-memory, declarative-memory, and working-memory skills and adult native English speakers’ novel sound-category learning. Participants completed a sound-categorization task that required integrating two dimensions: one native (vowel quality), one non-native (pitch). Similar information-integration category structures in the visual and auditory domains have been shown to be best learned implicitly (e.g., Maddox et al., 2006). Thus, we predicted that individuals with greater procedural-memory capacity would better learn sound categories, because procedural memory appears to support implicit learning of new information and integration of dimensions. Seventy undergraduates were tested across two experiments. Procedural memory was assessed using a linguistic adaptation of the serial-reaction-time task (Misyak et al., 2010a,b). Declarative memory was assessed using the logical-memory subtest of the Wechsler Memory Scale-4th edition (WMS-IV; Wechsler, 2009). Working memory was assessed using an auditory version of the reading-span task (Kane et al., 2004). Experiment 1 revealed contributions of only declarative memory to dimensional integration, which might indicate not enough time or motivation to shift over to a procedural/integrative strategy. Experiment 2 gave twice the speech-sound training, distributed over 2 days, and also attempted to train at the category boundary. As predicted, effects of declarative memory were removed and effects of procedural memory emerged, but, unexpectedly, new effects of working memory surfaced. The results may be compatible with a multiple-systems account in which declarative and working memory facilitate transfer of control to the procedural system.

## Introduction

Learning new languages becomes increasingly difficult as we age. Individuals that begin learning a language after the age of seven are significantly less likely to attain native-like proficiency in that new language, for syntax and morphology (Newport, 1990) as well as for speech-sound perception and pronunciation (Flege, 1995, 1999; Díaz et al., 2012). It is, of course, impossible to equate the real-world language-learning context across infant and adult learners. However, highly controlled experimental studies have shown learning advantages for infants over adults for the same language structure (e.g., Gerken and Knight, 2015, finds infants learn a type of rule not learned by adults in Moreton et al., 2015; see also Gerken et al., unpublished). It has been suggested that the infant brain is particularly well suited for language learning (Newport, 1990; Goldowsky and Newport, 1993; Thompson-Schill et al., 2009). However, there is no consensus about the neural mechanisms underlying this developmental or maturational difference. This project focuses on two major factors that may account for variation in adult L2-learning outcomes: native-language (L1) biases and reliance on declarative vs. procedural memory systems. We relate individual differences in these memory-skill domains to sound-category learning, one crucial aspect of L2 learning that exhibits wide individual variation for adult learners.

Adults’ experience with their native language leads to L1 biases that inhibit their ability to process and learn second-language (L2) speech-sound contrasts (Flege, 1995, 1999; Best et al., 2001; Iverson et al., 2003; Lotto et al., 2004; Best and Tyler, 2007). These biases hinder L2 learning and phonological processing (Lively et al., 1993, 1994; McCandliss et al., 2002; McClelland et al., 2002; Lotto et al., 2004; Lim and Holt, 2011; Gabay and Holt, 2015; Gabay et al., 2015). However, they likely result in better processing efficiency in the native language (Zhang et al., 2005; see also Kuhl et al., 2005).

The second factor that we argue contributes to the disparity in language learning (including sound-category learning) between adults and infants is differences in reliance on two different memory systems: the procedural-memory system (which subserves implicit learning) and the declarative-memory system (which subserves explicit learning). Declarative memory supports conscious recall of facts and events and can store such information for years (Knowlton and Squire, 1996; Ullman, 2004; Newman et al., 2010; Lum et al., 2012; see also Ullman and Pierpont, 2005). Learning occurs primarily explicitly through this system and can be achieved following a single exposure, though it is strengthened by multiple exposures (Lum et al., 2012). Within language, declarative memory has been suggested to store the mental lexicon of memorized word-specific knowledge (Ullman, 2004). Declarative memory supports lexical knowledge by encoding, storing, and retrieving semantic knowledge (Eichenbaum, 2004; Squire, 2004). It is believed to be subserved by medial temporal lobe structures including the hippocampus (Squire, 2004).

While learning through the declarative-memory system is primarily explicit, learning through the procedural-memory system is primarily implicit. Procedural memory is less accessible to conscious awareness and enables gradual learning of habits and skills (Hayne et al., 2000), including sequencing, navigation, and probabilistic categorization (Lum et al., 2012). In language, the procedural-memory system is thought to support the learning and use of rule-governed aspects of grammar (Knowlton and Squire, 1996; see also Ullman, 2004; Ullman and Pierpont, 2005; Newman et al., 2010). Implicit learning of sequential regularities has been linked to an individual’s ability to use contextual and lexically predictive information when comprehending spoken language (Misyak et al., 2010a). Evidence of dissociations in lesion studies has led to the hypothesis that the procedural and declarative memory systems have distinct neural underpinnings (Reber, 2013). Procedural memory is believed to be subserved by the striatum, including the caudate nucleus (Squire, 2004; though see Carpenter et al., 2016).

In addition to the above-mentioned roles of procedural and declarative memory in language learning, working memory has also been demonstrated to make important contributions to language learning and processing. Working memory plays an important role in understanding and learning language by maintaining information in a short-term buffer while it is being processed (Lum et al., 2012). The phonological loop, a component of working memory, encompasses a phonological store and a rehearsal process, and facilitates the learning of phonological forms of new words (Baddeley et al., 1998; see also Baddeley and Hitch, 1974; LaBerge and Samuels, 1974). Reading-comprehension performance—specifically, retrieving facts and computing pronominal references—has been linked to working-memory capacity (Daneman and Carpenter, 1980). Evidence suggests that working memory is closely related to declarative memory; the prefrontal structures that foster information retrieval from declarative memory also support working memory (Buckner et al., 1999; Botvinick et al., 2001; Simons and Spiers, 2003).

There is growing interest in the idea that certain aspects of language are best learned implicitly/procedurally (Evans et al., 2009; Quam et al., 2015). Statistical learning, believed to underpin much of early language learning, has been linked to implicit learning (Gómez, 2016). Infants rely more heavily on implicit/procedural learning than explicit/declarative learning, because the neural structures that support implicit learning mature relatively early in typical development, while those that sustain explicit learning are slower to develop, undergoing significant maturation through 10 months of age (Jones and Herbert, 2006; Richmond and Nelson, 2007). Thus, the formation of memories in infants, an essential underpinning of learning, is largely unconscious and implicitly driven. Infants’ reliance on implicit learning and sparse native-language experience may result in flexibility about which dimensions are relevant to a language-learning task (Namy and Waxman, 1998; Woodward and Hoyne, 1999; Namy, 2001; Singh et al., 2013; Hay et al., 2015), facilitating the learning of new linguistic structures. By contrast, adults’ over-reliance on explicit-learning strategies (Filoteo et al., 2010) and their native-language biases (Flege, 1995; Best et al., 2001; Best and Tyler, 2007) may interact to produce rigidity in attending to and integrating unfamiliar dimensions when learning new categories (Quam et al., 2015).

Models of impaired language like the Procedural Deficit Hypothesis suggest that procedural deficits are predictive of poor language-learning outcomes (Ullman and Pierpont, 2005; Kemény and Lukács, 2010; Hedenius et al., 2011; Lum et al., 2012; but see Gabriel et al., 2011). In a recent paper, Morgan-Short et al. (2014) investigated how individual differences in memory skills affect learning of L2 syntax. At early stages of acquisition, they found relationships between declarative-learning ability and syntactic development, whereas at later stages of acquisition, they found relationships between procedural-learning ability and syntactic development.

To address the question of whether differences in L2 learning outcomes could be explained by individual differences in procedural-memory capacity (and/or by working-memory or declarative-memory capacity), the present study investigates learning of sound categories. We focus on sound categories because they are complex and defined over multiple dimensions (Holt and Lotto, 2006). In order to process speech effectively, various acoustic dimensions must be integrated and weighted appropriately to recognize each sound and each word. These aspects of speech-sound learning present opportunities to extend theoretical and methodological approaches from the visual category-learning literature (particularly the COVIS model—see descriptions below) to test the Procedural Deficit Hypothesis for language learning. There have been several recent extensions of approaches from the visual category-learning literature (in particular, rule-based vs. information-integration category paradigms) to speech-sound-category learning (Wade and Holt, 2005; Goudbeek et al., 2009; Maddox et al., 2013; Maddox and Chandrasekaran, 2014; see also Moreton et al., 2015), but none have linked category learning to individual differences in memory skills.

Much of the evidence that adults’ reliance on explicit-learning strategies impairs their learning of new categories comes from the visual-category-learning literature. The COVIS model of category learning (Ashby et al., 1998) assumes competition between two category-learning systems, an explicit, verbal (or “reflective”) system, and an implicit (or “reflexive”) system. In a line of research testing the COVIS model (e.g., Waldron and Ashby, 2001; DeCaro et al., 2008; Filoteo et al., 2010), adults have been taught two different types of category structures. The first is rule-based category structures, designed so that the distinction between the categories is verbalizable, or at least available to conscious awareness (e.g., thick bars vs. thin bars; bars that tilt to the left vs. bars that tilt to the right). The declarative-memory system, which relies on working memory and attention, has been argued to mediate rule-based category learning (Ashby et al., 1998; Filoteo et al., 2010).

The second type of category structure used by Ashby et al. (1998) is termed “information-integration” categories. These category structures are always defined along at least two dimensions, and integrating the dimensions is required for successful learning. In contrast to rule-based structures, information-integration structures are designed so that the ideal response strategy is not easily verbalizable. The procedural-memory system has been argued to mediate information-integration category learning.

Adults, who have mature declarative-memory systems, tend to over-rely on explicit-learning strategies, which are optimal for rule-based category learning, but not for information integration (Filoteo et al., 2010). According to the COVIS model, the two systems compete during learning, with one system eventually seizing control of the response (Ashby et al., 1998). Adults often show an initial bias toward using explicit-learning strategies and unidimensional rules (Shepard et al., 1961; Bruner et al., 1962). Over the course of training, some adults successfully shift to implicit/multi-dimensional strategies, while others persist in sub-optimal, unidimensional strategies (Smith et al., 2010; Maddox et al., 2013).

Experimental interventions can sometimes shift adults to the optimal, multi-dimensional strategy earlier in learning. For example, Filoteo et al. (2010) found that adults integrated two dimensions to learn categories more effectively if their access to explicit learning was blocked by taxing working memory (see also Maddox and Ing, 2005; Smith et al., 2014). Individuals with elevated depressive symptoms, associated with suppressed declarative memory, have also been shown to better learn information-integration categories than individuals without elevated depressive symptoms (Maddox et al., 2014).

According to the original COVIS model of dimensional integration in category learning, working memory, given its strong relationship to declarative memory, should also be inversely correlated with success in information-integration tasks (Ashby et al., 1998). However, given its important roles in language learning, and somewhat diverse findings on the impact of working memory in information-integration tasks since the original COVIS model (e.g., Lewandowsky et al., 2012), it could either facilitate or impair information integration in an auditory task like the one used here.

### The Present Study

Across two experiments, we taught healthy adults an information-integration sound-category structure and then related category-learning outcomes to individual differences in memory skills. The novel sound categories presented to participants varied along a phonologically non-native dimension, pitch, and a native dimension, vowel quality (second-formant frequency; F2). Optimal learning required integrating information from both cues. Because of the role procedural memory putatively plays in infant language learning, and based on evidence from prior category-learning work (Maddox and Ing, 2005; Filoteo et al., 2010; Maddox et al., 2014; Smith et al., 2014), we predicted that adults with stronger procedural-memory skills would better integrate the two acoustic dimensions. However, we also assessed learners’ declarative-memory skills and working-memory skills. Recent follow-ups to the original COVIS model (Erickson, 2008; Ashby and Maddox, 2011) have indicated contributions of multiple systems to category learning, and similar updates have been suggested for the Procedural Deficit Hypothesis for language learning (Lum and Conti-Ramsden, 2013; Kuppuraj et al., 2016). Thus, strong declarative- and working-memory skills could facilitate shifting from a suboptimal dimensional integration strategy to an optimal strategy.

## Experiment 1

### Materials and Methods

#### Participants

All study procedures for Experiment 1 were approved by the Institutional Review Board (IRB) Committee at the University of Arizona and all participants provided written informed consent. Twenty-nine undergraduates from the University of Arizona who were native speakers of English and over the age of 18 were recruited from the Psychology participant pool and participated for course credit in one 2-h session. When participants (occasionally) required more than 2 h to complete the study and were willing to stay, they were paid $5/half hour to complete the session. We aimed to recruit a diverse sample of healthy adults.^1^ Because of our interest in individual differences in language acquisition (and because, e.g., attention-deficit (hyperactivity) disorder, or AD(H)D, is highly comorbid with developmental language disorder), we did not exclude participants on the basis of a diagnosis of AD(H)D (Fidler et al., 2011). We also included participants with exposure to other languages as long as they were native speakers of English. Seven additional participants were tested but excluded from analyses: 5 because they did not complete all the experimental tasks, and 2 because they did not click in the correct (right-most) portion of the screen in any trials in the procedural prediction task.

**FIGURE 1:**
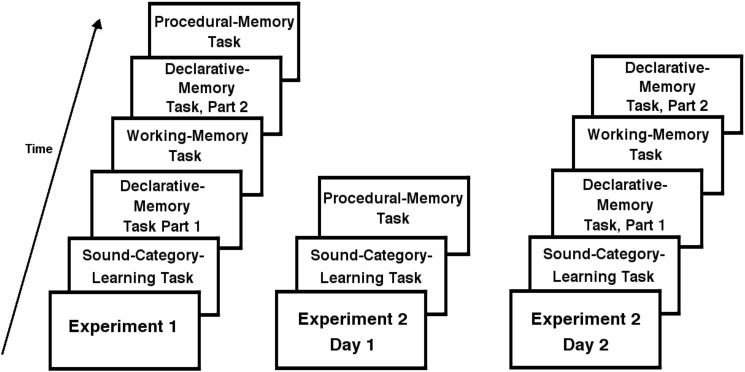
Sequence of events for Experiments 1 and 2. Experiment 1 was completed on 1 day, while Experiment 2 was completed over 2 days.

The session began with the sound-category-learning task, which took roughly 30 min. The first portion of the declarative-memory assessment came next, consisting of exposure and immediate recall. Participants next completed the working-memory assessment, which took on average 16 min, and then completed the delayed recall portion of the declarative-memory assessment (the entire declarative-memory assessment took approximately 15 min). Finally, they completed the procedural-memory assessment, which took roughly 30–40 min. **Figure 1** depicts the order of tasks in Experiment 1 (as well as each day of Experiment 2).

#### Sound-Category-Learning Task

##### Materials

Auditory stimuli were isolated vowels synthesized using Klatt (Klatt and Klatt, 1990), a speech synthesizer implemented within the Praat phonetic software program (version 5.3.43; Boersma and Weenink, 2008; Weenink, 2009). Sounds were synthesized at a uniform maximum amplitude of 70 dB SPL (see Weenink, 2009, for details on the voicing amplitude tier in KlattGrid), 0.4 (s) in duration, and contained two features to increase their naturalness. First, we inserted a pitch declination: between 0.25 and 0.3 s, the pitch gradually decreased to 96% of the original pitch height, then stayed at that value for the last 0.1 s. Second, we inserted an amplitude ramp at the end of the sound (using a custom Matlab script written by Sarah Creel), so that the amplitude declined linearly from 70 dB SPL to zero amplitude over the course of 10 ms, rather than clipping off at a higher amplitude.

**FIGURE 2:**
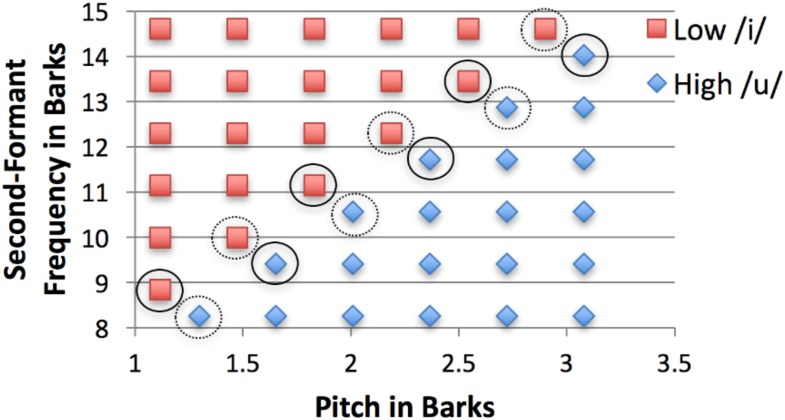
Synthesized speech stimuli varied in pitch (F0) and second-formant frequency (F2). Solid and dashed circles indicate two different sets of stimuli along the category boundary that were presented at the start of each training day in Experiment 2, but were intermixed with other stimuli in Experiment 1.

The 42 stimuli varied across two dimensions, pitch (F0) and vowel quality (second-formant frequency, or F2; see **Figure 2**). We intentionally included a native-language dimension (F2, used to differentiate the /i/ vs. /u/ vowels in English) and a non-native dimension (F0, which is not phonologically contrastive in English), to simulate L2 learning, in which some L2 dimensions might overlap with L1 and others will not. F0 ranged from 104 to 296 Hz; F1 was set to 448 Hz; F2 ranged from 1054 to 2366 Hz; and F3, F4, and F5 were set to 2722, 4019, and 4898 Hz, respectively. The ranges of F0 and F2 and the values of the other formants were modeled on recordings of the first author’s vocal range (a female, native-English speaker). F2 values were intended to range from an exaggerated /u/ to an exaggerated /i/ vowel. On the F0 and F2 dimensions, the stimuli were equally spaced along the Bark scale, a logarithmic scale designed to mimic frequency encoding in the human auditory system (Zwicker, 1961). Two categories were designed that differed equally on both dimensions. They could be roughly described as “high /i/” and “low /u/” categories, based on their centroids, but note that each category contained stimuli that spanned the full extent of each auditory dimension. Thus, the verbal descriptors “high /i/” and “low /u/,” if used as a strategy in the task, would not lead to high accuracy. Instead, to achieve high performance in the task, participants had to learn over training trials where to place the diagonal boundary between the categories.

##### Procedure

**FIGURE 3:**
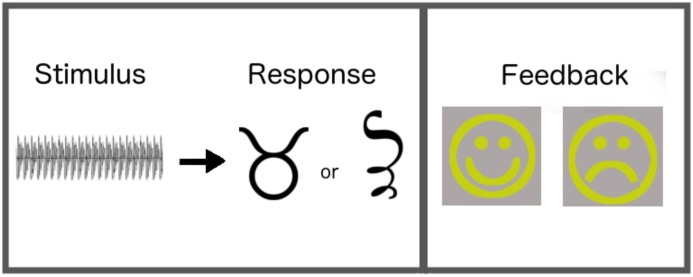
Within-trial sequence for each category-learning trial.

The experiment and all three memory assessments were programmed in the PsychoPy software program (version 1.79.00; Peirce, 2007) and administered on Mac Mini computers running Yosemite. Participants completed six training blocks, each of which presented all 42 synthesized stimuli in random order within each block. Participants sat in front of the computer in a soundproof testing room and were instructed to categorize each sound they heard through headphones (Sennheiser HD 280 PRO) to the best of their ability. In each trial, participants listened to an auditory stimulus, then responded by pressing one of two keys on the keyboard. The response keys were labeled with two unfamiliar symbols (see **Figure 3**).

Following each response, participants were provided with either positive or negative feedback, based on their answers. If participants correctly categorized the sound, then a large smiley face appeared (in yellow font; see **Figure 3**). If participants incorrectly categorized the sound, then a large frowny face appeared. We used smiley and frowny faces so that the same feedback could be used with preschool children in related studies (Quam et al., 2015, unpublished). The timing of presentation of feedback was based on prior work (Filoteo et al., 2010); it appeared immediately after the participant’s response and stayed on the screen for 500 ms. Immediate feedback has been shown to promote integration of multiple dimensions in category learning (Maddox and Ing, 2005). As training trials involved key-press judgments of category membership, we were able to use them to assess learning outcomes (e.g., by evaluating accuracy in block 6), rather than having to include separate test blocks. This enabled us to maximize training time, and thus increase the likelihood that participants would shift to an information-integration strategy by the end of the task.

#### Declarative-Memory Assessment

##### Materials

The logical-memory subtest of the Wechsler Memory Scale-4th edition (WMS-IV; Wechsler, 2009) was administered to participants to measure declarative-memory skills. Materials were purchased from Pearson-Clinical and adapted for computer administration. They consisted of two three-sentence-long fake news stories (one with a male protagonist, one with a female protagonist), yes/no questions about the stories, and a scoring rubric for evaluating the accuracy of participants’ recalled details about the stories. The first author (a female, native-English speaker) recorded each story. Each recording was 25 s long. Transcriptions of the stories can be found in the Wechsler Memory Scale-4th edition.

##### Procedure

Participants were instructed to pay as much attention as possible to an auditory reading of a short news story. After a recording of the story was played over headphones, a subsequent screen asked participants to type the story as exactly as possible into a dialog box. Participants then repeated this procedure for the second story. Following the completion of the immediate paragraph-recall task, participants completed the working-memory assessment, which took 20–30 min, depending on the participant’s speed.^2^ Participants next completed the declarative delayed paragraph-recall task: they were asked to recall the same stories again and enter their responses into dialog boxes without any reminder cues. They then answered “yes” or “no” to a series of questions assessing their memory for the content of each story. The responses of participants were individually analyzed and given quantitative results based on the Wechsler Memory Scale-4th edition response booklet from Pearson-Clinical. In the statistical analyses reported in the Results, we used delayed paragraph recall as the declarative-memory predictor, because it has been shown to bear a particularly strong relationship to hippocampal function (Gorwood et al., 2008). However, across the two experiments, delayed recall was highly correlated with both immediate paragraph-recall [for Experiment 1, (r(27) = 0.89, *p* < 0.001); note that because of the strong correlation in Experiment 1, immediate-recall scores were not coded for Experiment 2, because coding was time-intensive] and with yes/no question accuracy [r(68) = 0.60, *p* < 001].

#### Working-Memory Assessment

##### Materials

We employed an auditory version of a reading-span test of working memory (Kane et al., 2004), designed to quantitatively measure working memory (Daneman and Carpenter, 1980) by engaging participants in two concurrent tasks: semantic plausibility judgments and letter recall. While in previous reading-span tasks participants recalled a whole word, we asked participants to recall letters that were presented after each sentence in the sentence-judgment task. Both the auditory presentation and the use of letter recall instead of word recall were intended to reduce the impact of literacy skills on the task (Kane et al., 2004).

##### Procedure

Participants first completed training trials to learn the procedure of memorizing sequences of letters that were played over headphones, and then entering the letters into a dialog box in the correct order. The options for letters were listed at the top of the dialog box, with participants entering “NA” if they had forgotten one of the letters. The options were “h”, “j”, “k”, “l”, “n”, “p”, “q”, “r”, “s”, “t,” “y,” or “NA.” Participants completed six practice trials of responding to auditory sentences. Each sentence was either semantically plausible (“correct”) or implausible (“incorrect”), with participants indicating *correct* with the up arrow key and *incorrect* with the down arrow key. Participants then practiced the combined sentence/letter task, during which they heard three sequences that each contained three sentences, each followed by a single letter. Participants were instructed to respond *incorrect* or *correct* to each sentence as accurately and quickly as possible; their current accuracy percentage was displayed in the top right-hand corner to motivate them to keep their sentence-judgment accuracy above 80%. This was important to ensure that the letter-recall task was tapping working memory. Were participants to ignore the sentence-judgment task, they would not be balancing the letter-recall task with a concurrent task, which is necessary in dual-task paradigms (like the letter-recall task) to ensure that the task is tapping working-memory skills as designed (Daneman and Carpenter, 1980; Kane et al., 2004). At the end of each sequence, they were prompted to enter the letters at the end of the sentences into a dialog box.

After practicing the three sequences, participants completed the main task, which consisted of ten sequences (each sequence containing three sentences, three letters, and one dialog box for entering responses). Order of presentation of sentences and letters was randomized throughout the assessment. Unlike in previous uses of this method (e.g., Daneman and Carpenter, 1980; Kane et al., 2004), the set size of to-be-recalled letters did not vary (e.g., between 2 and 5 letters) but was fixed at 3 for all 10 sequences.

We calculated overall sentence accuracy to verify that participants were attending to both tasks. All participants had sentence-accuracy scores above 70%, indicating that the task was tapping working-memory skills as designed. In the statistical analyses reported in the Results, we used letter-recall accuracy as the working-memory predictor. Letter-recall accuracy was computed within each trial (i.e., a trial was correct only if all three letters were entered in the correct order).

#### Procedural-Memory Assessment

##### Materials

**FIGURE 4:**
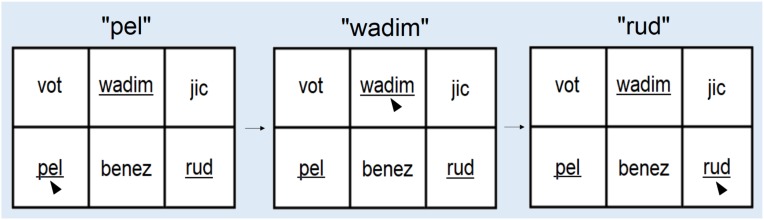
Example trial showing the grid of word stimuli displayed on the computer screen. The cursors (added for emphasis) point to the target string in each third of the trial (*pel, wadim, rud*, underlined for emphasis). The other three words are foils (*vot benez jic*).

We measured procedural-memory skills using a verbal adaptation (Misyak et al., 2010a,b) of the serial-reaction-time task (SRT). We chose a linguistic version of the SRT rather than a more traditional, visual SRT (in which, e.g., a dot appears in the four screen quadrants following a predictable pattern; Robertson, 2007) because we are interested in the link between procedural memory and language learning. Participants were exposed to visual-auditory strings of three non-words belonging to an artificial, non-adjacency language developed by Gómez (2002). Strings had the form *aXd*, *bXe*, and *cXf*, with ending non-words (*d, e, f*) dependent on beginning non-words (*a, b, c*). The dependency was non-adjacent because of the variable intervening item, which was sampled from a set of 24. Beginning and ending non-words were monosyllabic (beginning words *a, b*, and *c* were *pel, dak*, and *vot;* ending words *d, e*, and *f* were *rud, jic*, and *tood*). The set of 24 middle *X* items were bisyllabic (*wadim, kicey, puser, fengle, coomo, loga, gople, taspu, hiftam, deecha, wamey, skiger, benez, gesnim, feenam, laeljeen, chila, roosa, plizet, balip, malsig, suleb, nilbo*, and *wiffle*). A female English speaker produced auditory word tokens of the non-word items (Gómez, 2002^3^). Written forms of the non-words were presented on the screen in a 2 × 3 grid. The leftmost column of the grid contained only the beginning non-word items of the string (sampled from the set [*a*, *b*, *c*]), the center column contained the middle *X* tokens (sampled from the set [*X_*1*_…X_*24*_*]*)*, and the rightmost column contained only the ending non-word items of the string (sampled from the set [*d, e, f*]). **Figure 4** shows the grid of word stimuli from an example trial, with underlining added to show the 3 target words. The same visual grid accompanied all 3 auditory stimuli, and participants were meant to click on the visual word matching each of the auditory words (cursors indicate where the participant should click in response to each of the 3 auditory words). In this example, “pel” and “vot” are potential initial-string non-word elements, displayed in the left column; “wadim” and “benez” are potential middle-string elements, displayed in the middle column; “jic” and “rud” are potential final-string elements, displayed in the right column. Positions of targets and foils were pseudo-randomized and counterbalanced so that each appeared equally often within upper and lower positions.

##### Procedure

The procedure was modeled after that used by Misyak et al. (2010a,b). Participants were first presented with six training blocks of 72 unique 3-word strings (24 X-elements crossed with 3 dependency-pairs), for a total exposure to 432 strings. Each trial began by displaying the 2 × 3 grid of non-word tokens. After 250 milliseconds (ms.), participants heard the first non-word over headphones. Participants then used a computer mouse to click on the written word that matched the spoken word, with instructions emphasizing both speed and accuracy. The second and third non-words were played immediately after the previous response was registered. Following the third response, the screen cleared and a new set of non-words appeared 750 ms later. Each non-word occurred equally often (within a column) as a target and as a foil, preventing participants from anticipating which was the target and which was the foil for the initial and middle items. However, if participants learned the non-adjacent dependencies, they should subconsciously anticipate the third non-word based on its relation to the first non-word.

After exposure to the six training blocks, participants were presented with a test phase of 24 strings of ungrammatical non-words, with endings that violated the non-adjacent dependency that participants learned during the training blocks. A final recovery block of 72 grammatical strings, similar to the training blocks, followed the testing block. Participants were not notified of the transitions between blocks. To measure the degree of learning of non-adjacency patterns, participants were presented with a final prediction task. Participants were told that there were rules governing the sequencing of non-words in the auditory stimuli and were asked to identify the final target non-word in 24 stimulus strings upon being cued with only the first two non-word elements. Each trial in the prediction block began like training trials—each of the first two non-words was presented auditorily, and participants clicked the corresponding word on the screen. However, the third word was not presented auditorily, so participants had to guess which word would grammatically complete the string. Prediction-task accuracy was calculated as the percentage of trials with correct responses (computed over only trials where participants clicked in the right-most portion of the screen, so that chance performance is 50%). In the statistical analyses reported in the Results, we used prediction-task accuracy as the procedural-memory predictor, because this measure had been used in prior work to investigate individual differences (Misyak et al., 2010a).

#### Model-Based Analyses

Next we applied a series of computational models, used in many previous studies, to identify the category-learning strategy each participant employed in each learning block. The output of the modeling procedure was the best-fitting model for each participant in each of the 6 training blocks. We then computed the “Number of Linear Blocks:” the number of training blocks (out of 6 in Experiment 1 and 7 in Experiment 2) in which each participant’s categorization responses were best fit by either a sub-optimal linear category boundary (or “GLC,” for “general linear classifier”; indicating integration of dimensions but a category boundary that is offset from the true boundary) or an optimal linear boundary (“OPT”).^4^ Thus, a higher number of linear blocks indicates greater integration of the two dimensions and closer-to-optimal categorization performance.

In the “Results” section, we include Number of Linear Blocks as a dependent measure in multiple-linear-regression models (alongside accuracy in the 6th training block). Below, we provide specific details on the modeling procedures (additional details are available in several previous papers, e.g., Maddox and Ashby, 1993; Maddox, 1999; Maddox et al., 2016; Noh et al., 2016).

Five types of models were included. Each model was fit to each participant’s responses in each training block, and the best-fitting model was selected using the Bayesian information criterion (BIC; Kass and Wasserman, 1995). BIC is defined as:

$${\mathrm{BIC}}_{i}=2\ln L_{i}+\ln(n)V_{i}$$

where *L_i_* is the likelihood for model *i*, *V_i_* is the number of free parameters in the model, and *n* is the number of trials in each block. Notice that BIC penalizes models with more free parameters. Smaller BIC values indicate a better fit to the data. The best fitting model was defined as the model with the smallest BIC value.

The first model assumed random responding (RR). The second model assumed that the participant used a unidimensional rule using the X dimension, F0 (UDX). The third model assumed a unidimensional rule based on the Y dimension, F2 (UDY). The fourth model assumed a diagonal but sub-optimal linear decision criterion (general linear classifier; GLC). Note that only GLC models with positive slopes were accepted as best-fitting models, as negative slopes would not truly indicate dimensional integration (since the dimensions are integrated backward^5^). Finally, the fifth model assumed that the participant used the optimal, diagonal linear boundary (OPT). All of these analyses were replicated with the Akaike information criterion (AIC, Akaike, 1974) that is also based on maximum-likelihood estimation procedures but uses a different penalization equation. The pattern of results mirrored those for BIC. These results will not be discussed further.

### Results

We conducted multiple-linear-regression models for each outcome measure, to simultaneously consider the impact of procedural-, declarative-, and working-memory skills on category-learning outcomes. As described in the Methods, we chose *a priori* to measure procedural skills by analyzing accuracy in the prediction task, and to measure declarative skills by analyzing delayed paragraph recall. For working memory, we analyzed letter recall accuracy, as is standard for the listening-span task (Kane et al., 2004). Means and standard deviations on these measures are listed in **Table 1**. None of the three memory-skill domains was significantly correlated with any other in either experiment (*r*’s < 0.25, *p*’s > 0.15).^6^

**Table 1:** Measures of memory skills.

Measure	Experiment 1		Experiment 2	
	Mean (*SD*)	Range	Mean (*SD*)	Range
Procedural prediction accuracy	0.63 (0.21)	0.38–1.00	0.58 (0.19)	0.38–1.00
Declarative delayed recall	22.16 (7.71)	8.00–41.00	18.65 (6.61)	4.00–35.00
Working-memory letter recall	0.90 (0.10)	0.67–1.00	0.87 (0.13)	0.44–1.00

We related the three memory-skill predictor variables to two measures of category-learning outcomes: (1) accuracy in the 6th and final training block (“Block 6 Accuracy”) and (2) total number of training blocks with a GLC or OPT best-fitting model (“Number of Linear Blocks”). Number of Linear Blocks ranged from 0 to 6. Below, we report regression analyses for each dependent variable in turn. For regression analyses, standardized coefficients betas and adjusted *R*^2^ values are reported throughout the paper.

#### Block 6 Accuracy

Accuracy increased significantly over the course of the 6 training blocks from 65.82 to 72.50% [t(29) = 2.68, *p* = 0.012]. Block 6 accuracy also significantly exceeded chance [50%; *t*(28) = 10.87, *p* < 0.001]. The multiple linear regression model including procedural prediction accuracy, declarative recall, and working-memory letter recall as predictors showed no significant effects (see **Table 2** for standardized coefficients betas for all regression models predicting Block 6 Accuracy across Experiments 1 and 2).

**Table 2:** Standardized coefficients betas for all factors in regression models predicting *Block 6 Accuracy*, for Experiments 1 and 2.

Factor	Experiment. 1	Experiment 2, Day 1	Experiment 2, Day 2
Procedural prediction accuracy	0.110	0.366	0.220
Declarative delayed recall	0.148	−0.045	0.132
Working-memory letter recall	−0.175	0.256	0.401

#### Number of Linear Blocks

**FIGURE 5:**
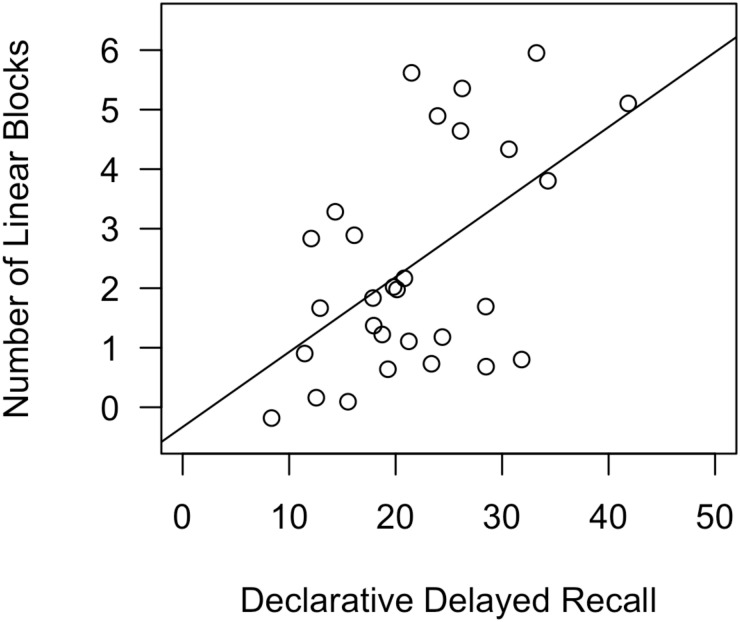
Scatterplot, with best-fit line, depicting the impact of declarative-memory skills on number of linear blocks in Experiment 1. In a regression analysis that also included procedural-memory skills and working-memory skills as predictors, declarative-memory skills were the only significant predictor.

On average, participants used a linear decision boundary (OPT or GLC) in 2.4 of 6 blocks. The regression model revealed a significant effect of declarative skills [β = 0.506, *t*(25) = 2.91, *p* = 0.008] on number of linear blocks. The model overall explained a significant proportion of variance in number of linear blocks [*R*^2^ = 0.213, *F*(3,25) = 3.53, *p* = 0.029]. **Table 3** reports standardized coefficients betas for all factors in all regression models predicting Number of Linear Blocks in Experiments 1 and 2. **Figure 5** depicts a scatterplot, with a best-fit line, for Number of Linear Blocks as a function of declarative-memory skills.

**Table 3:** Standardized coefficients betas for all factors in regression models predicting *Number of Linear Blocks*, for Experiments 1 and 2.

Factor	Experiment 1	Experiment 2, Day 1	Experiment 2, Day 2
Procedural prediction accuracy	0.069	0.210	0.412
Declarative delayed recall	0.506	0.005	0.019
Working-memory letter recall	0.091	0.386	0.289

### Discussion

In Experiment 1, regression models indicated that surprisingly, declarative memory significantly predicted the total number of linear blocks, a measure of cue integration in category learning. The effect of declarative memory is surprising and may indicate that not enough time or motivation was given for people to shift over to a procedural/integrative strategy. This possibility motivated Experiment 2, in which participants received twice the training (distributed over two training days) and the start of each training session focused on stimuli near the category boundary, to encourage dimensional integration. Beginning training with difficult training items that straddle the category boundary has been shown to improve learning of information-integration categories (Spiering and Ashby, 2008).

## Experiment 2

### Materials and Methods

#### Participants

All study procedures for Experiment 2 were approved by the IRB Committee at the University of Arizona and all participants provided written informed consent. Forty-one undergraduates from the University of Arizona were recruited from the Psychology participant pool, and participated for course credit, pay ($10/h), or a combination, in two 60- to 90-min sessions. Inclusion criteria and recruitment procedures matched Experiment 1. Twenty-four more participants were tested but excluded from analyses. This number exceeded exclusions in Experiment 1 mainly because of logistical issues involved with testing on 2 days; most excluded participants either did not complete both the category-learning task and the procedural task within the first 1-h test session (and could not stay to finish it for pay; 8) or did not return for the second session (5). The remainder were excluded for computer malfunctions (3), native languages other than English (4), evidence of current drug use (1), very poor performance on all tasks, including a very low score on the working-memory sentence judgment task (11% correct) that invalidated the procedure (1), and failing to click in the correct (right-most) portion of the screen in any trials in the procedural prediction task (2).

The two sessions were typically scheduled 2 days apart (due to scheduling constraints, two participants’ sessions were 3 days apart, and two participants’ sessions were 6 days apart). Both sessions began with the category-learning task, which took roughly 30 min. On day 1, the second task was the procedural-memory task. On day 2, the second task was part 1 of the declarative-memory task, followed by the working-memory task and part 2 of the declarative-memory task (see **Figure 1** for a diagram comparing the order of tasks between experiments).

#### Sound-Category-Learning Task

##### Materials

Materials for the sound-category-learning task were identical to Experiment 1.

##### Procedure

The procedure differed in two ways from Experiment 1, with the goal of promoting increased integration of the pitch and F2 dimensions in participants’ categorization strategies (i.e., more use of diagonal linear categorization boundaries, whether sub-optimal—GLC—or optimal—OPT). First, twice the training was given over the course of two different sessions. Second, while the same inventory of stimuli was presented on each day of Experiment 2 as in Experiment 1, the order of presentation differed. From each of the 6 original training blocks, 6 of the 12 stimuli that straddled the category boundary were pulled out of the block and moved to a “block 0” at the beginning of the task. The intention of block 0 was to highlight the category boundary by presenting only stimuli from along the boundary (see Spiering and Ashby, 2008, for a similar procedure). We hoped that presenting only boundary stimuli at the start of training would encourage learners to correctly categorize those stimuli, which would require learning precisely where the diagonal boundary was located in the category space and relying on both dimensions to do so. By contrast, when boundary stimuli were intermixed with non-boundary stimuli, a learner could achieve fairly high accuracy overall (as high as 85.7% correct) even if performing at chance (50% correct) on the boundary stimuli. Different sets of 6 “boundary” stimuli were pulled from blocks 1, 3, and 5 than from blocks 2, 4, and 6, so that across the entire training, each “boundary” stimulus occurred 3 times in block 0 and 3 times across blocks 1–6. **Figure 2** indicates the 2 sets of 6 “boundary” stimuli with solid and dashed circles, respectively. Instead of 6 blocks of 42 trials as in Experiment 1, the task on each day consisted of 7 blocks of 36 trials: block 0 (containing the difficult “boundary” stimuli), and blocks 1–6.

#### Memory Assessments

The procedural-memory, declarative-memory, and working-memory assessments were identical to Experiment 1.

### Results

For each training day, we conducted the same analyses as in Experiment 1. Means and standard deviations on each memory measure in each experiment are listed in **Table 1**. The below results are organized by training day. For each day, as for Experiment 1, we report regression analyses for each dependent variable in turn (Block 6 Accuracy and Number of Linear Blocks), relating them to procedural-, declarative-, and working-memory skills. The range of Number of Linear Blocks was slightly different in Experiment 2 than in Experiment 1. Number of Linear Blocks ranged from 0 to 7 instead of from 0 to 6, because of the inclusion of block 0 (as in Experiment 1, a score of 7 represents never using a linear strategy). Accuracy was also higher in Experiment 2 block 6 (on both days) than in Experiment 1 block 6, because in Experiment 2 block 6, half of the difficult “boundary stimuli” were pulled out into block 0.

#### Day 1

##### Block 6 accuracy

**FIGURE 6:**
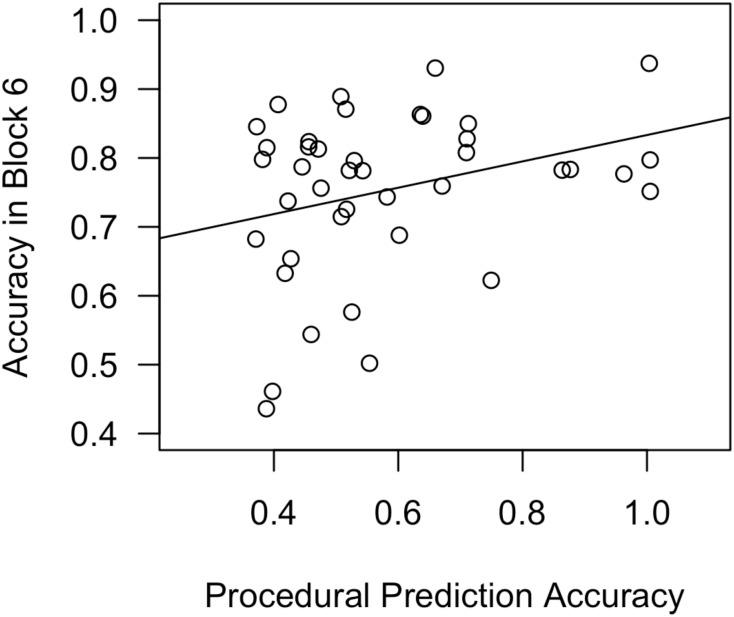
Scatterplot, with best-fit line, depicting the impact of procedural-memory skills on Block 6 accuracy in Experiment 2, day 1. In a regression analysis that also included declarative-memory skills and working-memory skills as predictors, procedural-memory skills were the only significant predictor.

On day 1, accuracy increased significantly between training blocks 1 and 6 from 57.38 to 75.34% [t(40) = 9.37, *p* < 0.001]. Block 6 accuracy also significantly exceeded chance [50%; *t*(40) = 13.65, *p* < 0.001]. The multiple-linear-regression model including procedural prediction accuracy, declarative delayed paragraph recall, and working-memory letter accuracy as predictors of block 6 accuracy showed a significant effect of procedural prediction accuracy [β = 0.366, *t*(37) = 2.28, *p* = 0.028]. However, the model overall did not explain a significant proportion of variance in accuracy scores [*R*^2^ = 0.083, *F*(3,37) = 2.20, *p* = 0.104].^7^ **Table 2** reports standardized coefficients betas for all regression models predicting Block 6 Accuracy. **Figure 6** depicts a scatterplot, with a best-fit line, for Block 6 Accuracy as a function of procedural-memory skills.

##### Number of linear blocks

**FIGURE 7:**
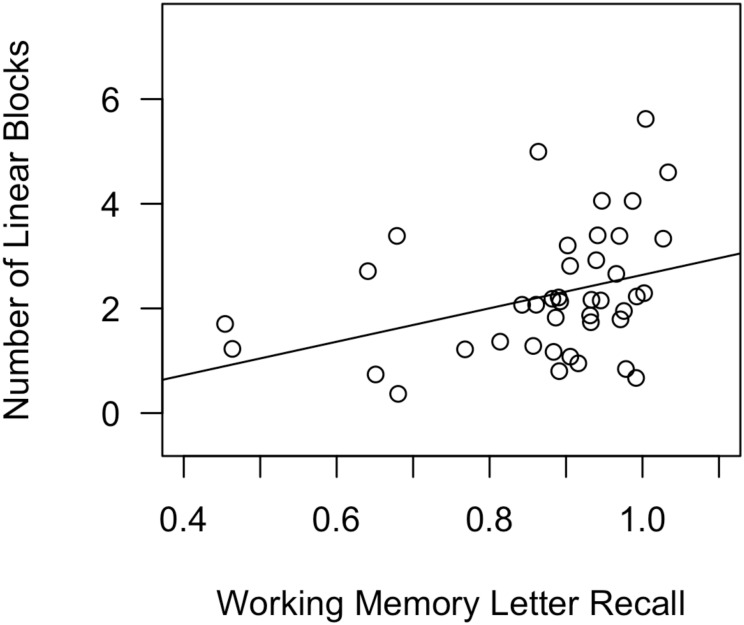
Scatterplot, with best-fit line, depicting the impact of working-memory skills on number of linear blocks in Experiment 2, day 1. In a regression analysis that also included declarative-memory skills and procedural-memory skills as predictors, working-memory skills were the only significant predictor.

On average, participants used a linear decision boundary (OPT or GLC) in 2.3 blocks (again, the possible scores ranged from 0–7). The regression model revealed that working-memory skills significantly predicted the number of blocks in which participants used a linear strategy [β = 0.386, *t*(37) = 2.48, *p* = 0.018]. However, the model overall did not explain a significant proportion of variance in number of linear blocks [*R*^2^ = 0.091, *F*(3,37) = 2.33, *p* = 0.089].^8^ **Table 3** reports standardized coefficients betas for all factors in all regression models predicting Number of Linear Blocks. **Figure 7** depicts a scatterplot, with a best-fit line, for Number of Linear Blocks as a function of working-memory skills.

#### Day 2

##### Block 6 accuracy

**FIGURE 8:**
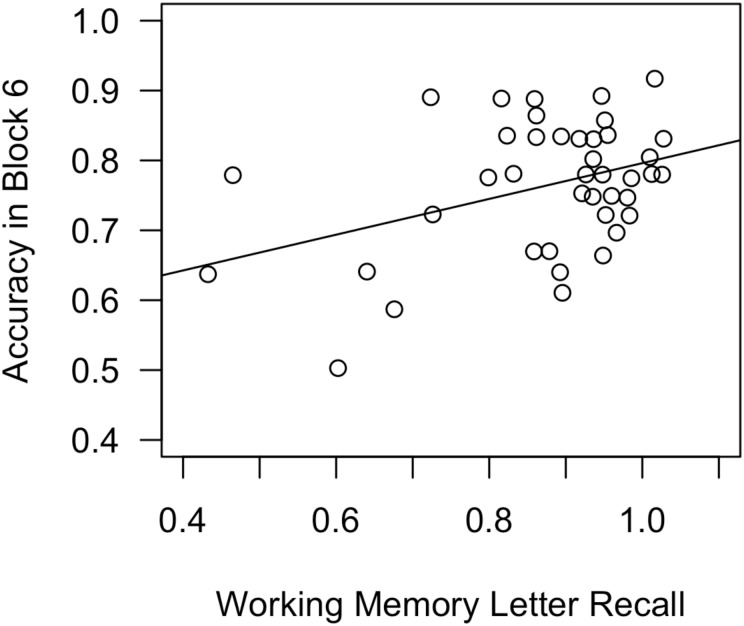
Scatterplot, with best-fit line, depicting the impact of working-memory skills on Block 6 accuracy in Experiment 2, day 2. In a regression analysis that also included declarative-memory skills and procedural-memory skills as predictors, working-memory skills were the only significant predictor.

On day 2, accuracy increased significantly between training blocks 1 and 6 from 71.75% to 76.42% [*t*(40) = 2.96, *p* = 0.005]. Block 6 accuracy also significantly exceeded chance [50%; *t*(40) = 17.96, *p* < 0.001]. However, block 6 accuracy on day 2 did not significantly exceed block 6 accuracy on day 1 (75.34%; n.s.), suggesting that participants may have already reached asymptotic accuracy on day 1. Block 6 accuracy on the 2 days was significantly correlated [*r*(39) = 0.69, *p* < 0.001]. For day 2, the multiple-linear-regression model showed that working-memory skills significantly predicted block 6 accuracy [β = 0.401, *t*(37) = 2.66, *p* = 0.012]. The model overall explained a significant proportion of variance in accuracy scores [*R*^2^ = 0.148, *F*(3,37) = 3.32, *p* = 0.030]. **Figure 8** depicts a scatterplot, with a best-fit line, for Block 6 Accuracy as a function of working-memory skills.

##### Number of linear blocks

**FIGURE 9:**
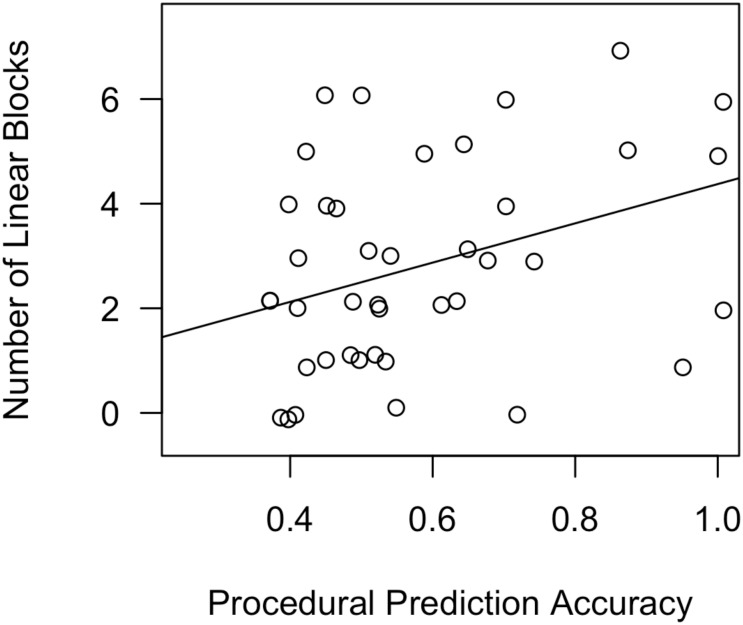
Scatterplot, with best-fit line, depicting the impact of procedural-memory skills on number of linear blocks in Experiment 2, day 2. In a regression analysis that also included declarative-memory skills and working-memory skills as predictors, procedural-memory skills were the only significant predictor.

On average, participants used a linear decision boundary (OPT or GLC) in 2.8 blocks. The regression model revealed that procedural prediction accuracy significantly predicted the number of linear blocks [β = 0.412, *t*(37) = 2.66, *p* = 0.012]. Working-memory skills did not reach the cutoff for statistical significance [β = 0.289, *t*(37) = 1.91, *p* = 0.064]. The model overall explained a significant proportion of variance in number of linear blocks [*R*^2^ = 0.143, *F*(3,37) = 3.22, *p* = 0.034]. **Figure 9** depicts a scatterplot, with a best-fit line, for Number of Linear Blocks as a function of procedural-memory skills.

### Discussion

Whereas Experiment 1 had revealed a significant effect of declarative memory on sound-category learning, Experiment 2 instead showed significant effects of working memory and procedural memory. On Day 1, in regression models, procedural memory significantly predicted accuracy (though note that the model did not explain a significant proportion of variance and the correlation between procedural memory and accuracy did not reach the cutoff for statistical significance), and working memory significantly predicted the total number of blocks in which participants used a two-dimensional, linear strategy (while the latter model did not explain a significant proportion of variance, the correlation was significant). On Day 2, regression models showed that procedural memory significantly predicted the number of linear blocks, while working-memory significantly predicted accuracy.

We had predicted stronger effects of procedural memory in Experiment 2 vs. Experiment 1, particularly on Day 2, when participants were given more time to shift to an implicit-learning strategy. Even on Day 1, we had expected that starting the training with the stimuli that straddled the category boundary (half of the difficult “boundary stimuli” were pulled out of training blocks 1–6 and presented in block 0) would encourage participants to use an implicit-learning strategy (i.e., to rely on procedural memory) rather than an explicit-learning (declarative-memory-based) strategy. However, the effects of working memory in Experiment 2 were surprising. It is not obvious which differences between the designs of Experiments 1 and 2 might account for the effects of working memory that emerged only in Experiment 2. It is possible that starting the training with the stimuli that straddled the category boundary somehow favored participants with strong working-memory skills in addition to strong procedural-memory skills, though the precise reason for this pattern is not clear.

## General Discussion

Across two experiments, healthy adult participants learned artificial sound categories and participated in assessments of procedural-, declarative-, and working-memory skills. Based on the COVIS model of category learning (Ashby et al., 1998) and the Procedural Deficit Hypothesis for language learning (Ullman and Pierpont, 2005; Kemény and Lukács, 2010; Hedenius et al., 2011; Lum et al., 2012), we had predicted that procedural skills would best predict learning outcomes for categories that require integrating two dimensions using a non-verbalizable strategy. Surprisingly, in Experiment 1, only declarative memory significantly impacted the number of training blocks in which participants integrated the cues. In Experiment 2, the trial orders were redesigned to draw attention to the category boundary and promote cue integration, and more time was provided to shift to an implicit-learning strategy, via a second day of training. Experiment 2 revealed significant effects of both procedural memory and working memory. On Day 1, procedural memory significantly predicted accuracy (though note caveats in the Experiment 2 Discussion, above), and working memory significantly predicted the total number of blocks in which participants used a two-dimensional, linear strategy. On Day 2, procedural memory significantly predicted the number of linear blocks, while working-memory significantly predicted accuracy.

The effects of procedural-memory skills on multidimensional-category-learning outcomes in Experiment 2 (where more time and encouragement were provided to support using implicit-learning strategies) provide support for our original prediction. This prediction was based on the Procedural Deficit Hypothesis (Ullman and Pierpont, 2005), as well as on the COVIS model for learning of information-integration category structures (Ashby et al., 1998). Several recent studies had also provided additional support for the link between procedural memory and dimensional integration. For example, learners with elevated depressive symptoms, which are associated with suppressed declarative memory, show better information-integration learning (Maddox et al., 2014). Reducing the time to process feedback before the next categorization trial also impairs rule-based but not information-integration category learning, arguably by reducing access to explicit strategies (Maddox et al., 2004). Recent work has also shown inverse relationships between working-memory skills and information-integration learning. Filoteo et al. (2010) found that taxing participants’ working memory using an interleaved number-recall task increased cue integration, putatively by blocking participants’ access to declarative strategies.

Nevertheless, multiple-linear-regression models also revealed facilitative effects on category learning of declarative memory in Experiment 1 and working memory in Experiment 2. These results are surprising considering our original prediction that procedural memory would uniquely predict sound-category learning in an information-integration task. In particular, the original COVIS model suggested that high declarative ability should be associated with poor information-integration learning (Ashby et al., 1998).

However, not all prior evidence supports the notion that declarative- and working-memory skills are inversely predictive of information-integration learning. Some studies have found that working memory is positively correlated with both rule-based and information-integration category learning. Lewandowsky et al. (2012) found that working-memory skills predicted both types of category learning, and predicted the ability to focus on task-relevant strategies, irrespective of whether the task required integrating multiple dimensions (see also Kalish et al., 2017). Craig and Lewandowsky (2012) found that working-memory capacity predicted category-learning speed but not the particular strategy participants used. Other studies have cast doubt on the notion of dissociable category-learning systems. In an fMRI study, Carpenter et al. (2016) found substantial overlap in the brain regions recruited for rule-based and information-integration tasks, including activation in the medial temporal lobes and hippocampus (see also Stanton and Nosofsky, 2013).

Even within the COVIS framework, more recent discussions have focused on system-level interaction, including the possibility that on some tasks it might be beneficial to flexibly switch between category-learning strategies trial-by-trial (Erickson, 2008; Ashby and Maddox, 2011). The process associated with switching between rule-based and information-integration types of strategies suggests that even highly skilled learners may not deploy solely procedural strategies. It could be that learners with strong declarative-memory skills or working-memory skills better manage the transition between explicit and implicit strategies. Thus, it could be that our results reflect the fact that learners with stronger procedural-memory skills are more successful at integrating multiple dimensions in category learning, but strong declarative- and working-memory skills are also beneficial in that they enable learners to efficiently shift over to an optimal cue-integration strategy. However, the fact that effects of declarative memory went away in Experiment 2 suggests that, when provided with enough time and support, participants shifted to greater use of implicit-learning strategies.

While the sound-category-learning task used here is ideal for testing how well the COVIS model generalizes to a language-learning task, it could be a less ideal test of the Procedural Deficit Hypothesis. The Procedural Deficit Hypothesis (Ullman and Pierpont, 2005) emerged out of a proposal that language grammar, particularly structure involving sequences (e.g., syntax or morphology) is best learned procedurally, while vocabulary is best learned declaratively (Ullman, 2004). Thus, it is not entirely clear whether this theoretical framework would predict that speech-sound learning is learned via the same route as syntax or morphology. The unsupervised way in which speech-sound categories in the brain emerge from experience with speech seems somewhat compatible with theoretical descriptions of statistical learning of language structure (Evans et al., 2009), which has been linked to implicit learning (Gómez, 2016). Nevertheless, extending approaches like the COVIS framework beyond speech-sound learning and into the domains of syntax and morphology could prove a more ideal test of the Procedural Deficit Hypothesis, and such extensions could be an important area for future research. One challenge for future work applying COVIS-type models to grammar learning will be to define the “dimensions” (analogous to the speech sound dimensions used here, pitch vs. second-formant frequency) for syntactic or morphological structures in order to relate dimensional integration to procedural-memory skills.

With regard to the present results, it must be noted that effects of working memory on category-learning outcomes could have been limited by ceiling effects on the working-memory task. Across the two experiments, 14/74 participants (19%) scored 100% on letter recall accuracy. The design of the working-memory task appears to have contributed to the ceiling effects we found, as only a set size of three sentence-letter combinations was used. This constant set size contrasts with prior work in which set sizes typically vary between two and five (or six) sentence-letter combinations (e.g., Daneman and Carpenter, 1980; Kane et al., 2004).

In addition, effects of procedural memory could have been limited by floor effects on the procedural prediction task. Across experiments, 27/70 participants (39%) scored at or below chance overall (50%). However, average procedural prediction accuracy (60%) is commensurate with prior work (61% in Misyak et al., 2010b), and ceiling and floor effects on the memory assessments did not preclude the extraction of meaningful findings. We still find significant effects of procedural- and working-memory skills. In future work, we expect that changes to the design of the working-memory task will reduce ceiling effects for working-memory accuracy, and may reveal stronger contributions of that predictor.

In conclusion, our findings suggest that individual differences in procedural memory predict accuracy and cue integration in an information-integration auditory category-learning task. This supports a model of category learning that argues that implicit skills predict cue integration (COVIS; Ashby et al., 1998). However, we also find evidence that both declarative memory and working memory predict category-learning outcomes. This result converges with other recent discussions of and follow-ups to the COVIS model (Erickson, 2008; Ashby and Maddox, 2011) that suggest contributions of multiple systems to category learning. In particular, it may be that strong declarative- and working-memory skills enable participants to efficiently switch away from a suboptimal, declarative, unidimensional strategy and toward an optimal, procedural, dimensional-integration strategy. However, much more work is needed to fully characterize the roles of each of these memory systems in category learning and cue integration as well as in language learning generally.

## Ethics Statement

This study was carried out in accordance with the recommendations of the University of Arizona Institutional Review Board with written informed consent from all subjects. All subjects gave written informed consent in accordance with the Declaration of Helsinki. The protocol was approved by the University of Arizona Institutional Review Board.

## Author Contributions

CQ conceived and designed the experiments, analyzed the data, and wrote the majority of the manuscript. AW conceived, designed, and performed the experiments and wrote portions of the manuscript. WTM analyzed the data using specialized modeling techniques and wrote portions of the manuscript. KG conceived and designed the experiments and wrote portions of the manuscript. AL conceived and designed the experiments and consulted on data analyses. All the authors discussed the results and implications and commented on the manuscript.

## Conflict of Interest Statement

WTM was employed by company Cognitive Design and Statistical Consulting, LLC.The remaining authors declare that the research was conducted in the absence of any commercial or financial relationships that could be construed as a potential conflict of interest.

## Footnotes

1 All research with undergraduate students runs the risk of recruiting a less diverse sample than the general adult population, in terms of age, socioeconomic variables, racial/ethnic variables, and cognitive and language skills. As a public university with relatively open admission criteria, the University of Arizona attracts a more representative undergraduate population than universities with lower admission rates. However, it is important to acknowledge the potential bias introduced in any research conducted with college students.

2 There is precedent for introducing an intervening task between immediate and delayed recall, and for the delay between the two tasks to vary depending on the length of the intervening task (e.g., Gorwood et al., 2008). In our Experiment 1 sample (where both immediate and delayed recall scores were tabulated), the average delayed recall score (21.79) was less than 1 point lower than the average immediate recall score (22.66). The strong correlation between immediate and delayed recall scores in Experiment 1 also indicates strong test-retest reliability across the two tasks. These facts provide some reassurance that the intervening working-memory task was not inappropriately cognitively taxing, nor was the delay inappropriately long. Across both experiments, the time between the onset of the working-memory task and the onset of the delayed recall task varied from 12 to 24 min (*M* = 16, *SD* = 3). The time between the onsets of the two tasks was not correlated with delayed recall performance [*r*(68) = −0.011, *p* = 0.929].

3 Stimuli used in Gómez (2002) were graciously shared by Rebecca Gómez.

4 We also calculated another dependent variable, “First Linear Block,” or the first training block in which each participant used a suboptimal or optimal linear decision boundary. However, Number of Linear Blocks and First Linear Block were highly correlated across Experiment 1 and Experiment 2, Day 1: [*r*(68) = −0.593; *p* < 0.001; a negative correlation was expected], and results were similar for both measures, so we have included only Number of Linear Blocks alongside Block 6 Accuracy.

5 In some previous applications of this modeling approach, conjunctive rule-based models were also applied to the data. These models assume that a unidimensional rule along the X dimension is combined with a unidimensional rule along the Y dimension. In the present case, these models are poorly distinguished quantitatively from the GLC and thus are excluded.

6 Note that across both experiments combined, procedural and declarative skills were significantly correlated [*r*(68) = 0.245, *p* = 0.041], but regression models were conducted separately for each experiment, so within-experiment correlations are more relevant for statistical models.

7 For comparison, we also computed the correlation between procedural prediction accuracy and block 6 accuracy, which did not reach the cutoff for statistical significance: *r*(39) = 0.30, *p* = 0.056.

8 For comparison, we also computed the correlation between working-memory skills and number of linear blocks, which was significant: *r*(39) = 0.342, *p* = 0.029.
